# The Impact of Community-acquired Pneumonia on Acute Exacerbation of Chronic Obstructive Pulmonary Disease Patients as Regards In-hospital Complications and Early Readmission

**DOI:** 10.2174/1874306402014010010

**Published:** 2020-04-09

**Authors:** Dina Ruby

**Affiliations:** 1 Chest Department, Ain Shams University, Cairo, Egypt

**Keywords:** Acute exacerbation, Chronic obstructive pulmonary disease, Community-acquired pneumonia, Length of hospital stay, Readmission, CRP

## Abstract

**Background and Objective:**

Pneumonia is a major reason for hospitalization for Acute Exacerbation of Chronic Obstructive Pulmonary Disease patients (AECOPD). There is limited data available on the outcomes of AECOPD patients with or without pneumonia. Therefore, the study investigates the prognosis of AECOPD patients with or without Community-acquired Pneumonia (CAP), concerning the Length of Hospital Stay (LOS), in-hospital complications and early readmission.

**Methods:**

This study was carried out on 100 male COPD patients without CAP, 90 patients with CAP who were admitted to the chest department of Ain Shams University hospital over a 1-year period. Data collection about LOS, in-hospital complications, was recorded and they were followed for 30 days to detect acute readmission.

**Results:**

The mean age was 64± 8 years old in COPD patients without CAP to 62± 12year old in patients with CAP, LOS in COPD patients with CAP was 11.30 ± 3.23 days to 7.57 ± 2.24 in patients without CAP, COPD patients with CAP had a higher rate of complications in comparison to those without CAP as 45.6%, 13% were admitted to Intensive Care Unit (ICU) respectively, 15.6%, 3% were mechanically ventilated respectively. LOS and C- Reactive Protein (CRP) were significant causes for readmission in COPD patients with and without CAP.

**Conclusion:**

COPD patients with CAP had longer LOS and more short term complications as ICU admission, mechanical ventilation and higher readmission rate in comparison to COPD patients without CAP.

## 1 INTRODUCTION

Chronic Obstructive Pulmonary Disease (COPD) is the fourth cause of death among chronic diseases, although it is preventable and treatable [1]. Acute Exacerbations of Chronic Obstructive Pulmonary Disease (AECOPD) are major events in COPD patient's life and are major causes of hospital admission and mortality [2]. As it accelerates, the lung function declines and worsens the prognosis of the disease [3].

Community-acquired Pneumonia (CAP) is a major cause of morbidity and mortality worldwide [4]. Pneumonia is the main reason for hospitalization for AECOPD and has poor outcomes [5]. Most of the patients with CAP had a high rate of Intensive Care Unit admission (ICU) and longer hospital stay than those patients without CAP [6].

There is still a lot of controversy about the survival or readmission rate of acute exacerbations of COPD with or without CAP, as some studies showed no discrepancy between acute exacerbations of COPD patients with or without pneumonia survival rate [7] and others found higher mortality rate among COPD patients with pneumonia [8, 9]. Therefore, we performed a prospective study to investigate the prognosis of acute exacerbations of COPD patients with or without CAP concerning the length of hospital stay, in-hospital complication (admission to an intensive care unit, use of mechanical, in-hospital mortality), and acute readmissions.

## 2 METHODS

### 2.1 Study Population

This prospective study was conducted in the Chest Department of Ain Shams University Hospital, Cairo, Egypt, after the approval of the local ethical committee from October 2018 and October 2019.

One hundred and ninety consecutive male patients ≥ 45 years old who were diagnosed with COPD, (according to the criteria established by the Global Initiative for Chronic Obstructive Lung Disease (GOLD), (1) and fulfilled the requirements of FEV_1_/Forced Vital Capacity (FVC) <70% following inhalation of a bronchodilator who were admitted in chest department ward at least for 24 hours, were enrolled in the study and divided into two groups according to targeted sample size calculation in each group [10]. The first group includes hundred COPD patients without CAP; the second group includes ninety COPD patients with CAP. The following patients were excluded; patients who had asthma, advanced malignancy, recent myocardial infarction (within 3months), renal insufficiency requiring hemodialysis, neurological diseases, and active tuberculosis or when no data on COPD diagnosis was available, and patients need ICU admission from the start.

The first group includes COPD patients with severe acute exacerbations, in which an exacerbation was defined as an increase in, or new onset of, ≥ 2 respiratory symptoms (sputum, cough, wheezing, dyspnea, and tightness of chest) with ≥ 1 symptom for more than 3 days, leading to initiation of treatment with systemic corticosteroids and/or antibiotics by the physician [11, 12].

The second group includes COPD patients with CAP, having a new pulmonary infiltrate on chest x-ray and 1 or more symptoms including cough, purulent sputum, positive auscul-tation or fever. The diagnosis of CAP was made on the basis of the American Thoracic Society/Infectious Disease Society of America guidelines [13].

Both groups were admitted to the ward of chest department and followed after discharge for one month to detect any readmission within 30 days following the index discharge.

### 2.2 Study Procedures

All patients were subjected to full medical history taking (age, pack /year, Comorbidities, long term oxygen therapy, medication before admission), physical examination, radio-logical investigations, laboratory investigations, arterial blood gases on room air (blood samples were collected within the first day of admission) and bacteriological investigations (sputum culture, sputum Ziehl–Neelsen). The Arabic language version of the COPD assessment test (CAT) questionnaire was used to assess the impact of COPD on the patient’s health [14]. The modified Medical Research Council (MMRC) dyspnea scale was also applied to asses dyspnea severity and all previous procedures were routinely done to all patients admitted to the chest department, either they were included or not in the study.

The data related to hospitalization were recorded including the length of hospital stay (defined as the difference between admission and discharge dates) and admission to an intensive care unit. The intensive care unit admission criteria for AECOPD were: unable to accommodate or failing to respond to noninvasive ventilator support, pH < 7.25, PaCO_2_ > 60 mm Hg, respiratory rate > 35 breaths/minutes, respiratory arrest, cardiovascular instability, impaired mental status, and persistent incapacity to remove respiratory secretions [15]. The criteria for intensive care unit admission for CAP were based on American Thoracic Society/Infectious Disease Society of America guidelines [13]. Data about the use of invasive mechanical ventilation, in-hospital mortality and early read-missions (defined as any readmission within 30 days following the index discharge) were also recorded, and also the previous data was recorded routinely in all patient's files.

Oral informed consent was obtained from all patients after explaining the aim of the study and that the study need the data from their files with no intervention to them.

### 2.3 Statistical Analysis

Statistical Package for the Social Sciences (SPSS) 20.0 (SPSS Inc.; Chicago, Illinois, united states) was used for statistical analysis. Chi-square or Fisher’s exact test was used for categorical variables and Student t or Mann-Whitney U test used for continuous variables. Odds Ratio (OR) and inde-pendent predictors of readmission were assessed by the logistic regression analyses. Descriptive statistics were expressed as mean value + standard deviation for continuous data and number (%) for categorical data. The value of p <0.05 was considered to be statistically significant.

## 3 RESULTS

During the study period, 220 patients were eligible for inclusion criteria, while thirty patients (20 patients with CAP and 10 patients without CAP) were lost during the follow-up periods.

Finally, a total of 190 COPD patients (100 patients without CAP and 90 patients with CAP) were enrolled. The mean age was 64 ± 8 years old in COPD patients without CAP to 62± 12year old in patients without CAP. There was no statistical difference between two groups regarding age, pack/year, the COPD assessment test questionnaire, modified Medical Research Council dyspnea scale, comorbidities, long term oxygen therapy and medication before admission, as shown in Table **1**, but regarding the length of hospital stay, there is a statistically significant difference between COPD patients with and without CAP (p<0.001).

Regarding arterial blood gases on room air and laboratory investigation in Table **2**, there is no statistical difference between COPD patients with or without CAP except regarding total leukocyte count, hemoglobin(55%, 35.6% of COPD patients without CAP, with CAP had anemia respectively) and C- Reactive Protein (CRP).

In Table **3**, COPD patients with CAP had a higher rate of complications in comparison to COPD patients without CAP as 45.6%, 13% were admitted to ICU, respectively, 15.6% of COPD patients with CAP were mechanically ventilated in contrast to 3% only of COPD patients without CAP. Therefore, there was a statistical difference between both groups with regards to complications (p<0.001). As regard to in-hospital mortality, two patients died, one with CAP and one without CAP.

Acute readmission in Table **4** was more evident in COPD patients with CAP as 47% of COPD patients with CAP were readmitted within one month of discharge and 27% only of COPD patients without CAP were readmitted with a stat-istically significant difference between both groups.

By using Multivariate logistic regression analysis for readmission in COPD patients without CAP in Table **5**, it was found that length of hospital stay and C- reactive protein are significant causes with readmission. In Table **6**, length of hospital stay, total leukocyte count, C- reactive protein, intensive care unit admission and mechanical ventilation were all significant causes with readmission in COPD patients with CAP.

## 4 DISCUSSION

COPD patients with CAP had poor outcomes, mainly in elderly such as morbidity, high mortality rate, increased length of stay in ICU, and invasive mechanical ventilation [16]. This study in addition to other aforementioned studies, we focused on short term complication and acute readmission rate in both COPD patients with and without CAP and it was found that COPD patients with CAP had a longer length of hospital stay and more short term complications as ICU admission, invasive mechanical ventilation and higher readmission rate.

Restrepol *et al*. [16] found that patients with pneumonia had poorer outcomes, both for mortality and a prolonged length of stay (longer than 7 days) when compared to patients with no evidence of pneumonia in COPD exacerbation. COPD patients with CAP had higher 30- and 90-day mortality than patients without CAP [11], which is similar to this study as the length of hospital stay in COPD patients with CAP was 11.30 ± 3.23 days to 7.57 ± 2.24 in COPD patients without CAP. Myint *et al*. [9] found that COPD exacerbations associated with the radiological finding of pneumonia have worse outcomes in terms of inpatient mortality(11% in COPD patients with pneumonia *vs*. 7% in COPD patients without pneumonia) and length of stay after taking into account potential confounding factors which were matched with this study as patients with CAP had a longer length of stay in hospital but different from them in the inpatient morality as there was no difference between both groups in our study as this may be due to small sample size in comparison to a large sample size of their study. Although there is an important point to highlight that the previous studies were carried in developed countries (United States and United Kingdom respectively), they had similar finding to this study which was carried in Egypt which reflects the clinical and economic burden of CAP on COPD patients in both developing and developed countries, but of course, the economic burden will be more on the developing countries.

Shin *et al*. [17] showed that the 180 day mortality was higher in AECOPD with CAP than AECOPD without CAP The readmission rate within 6 months was not significantly different between two groups, but it has been observed that readmission within 30 days after discharge was strongly related to the high mortality rate in both groups, especially in AECOPD with CAP; this was matched with this study as readmission is higher in AECOPD with CAP within 30 days after discharge but we did not follow patients for 180 days to assess mortality rate.

Earlier studies found that patients with pneumonic exacerbations tend to be old, had more Comorbidities, a longer hospital stay, and more assisted ventilation compared with patients with non-pneumonic exacerbations [8, 9, 18]. This was confirmed in this study even after adjusting all confounding factors as age, pack/year, comorbidities, medication before admission, long term oxygen therapy, the COPD assessment test questionnaire and modified Medical Research Council dyspnea scale.

Sogaard *et al*. [5] conclude that pneumonia is common in patients hospitalized with acute COPD exacerbations and is associated with increased health resource utilization and poor prognosis compared with patients with non-pneumonic exacerbations as the median hospital stay length was 4 days for patients without pneumonia and 7 days for patients with pneumonia. Mechanical or noninvasive ventilation was found 3.3% and 6.7% of patients without pneumonia respectively, compared with 6.9% and 9.7% of patients with pneumonia and readmission was more likely higher within 30 days following discharge in patients with pneumonia (18.2%) *vs*. (17.5%) in patients without pneumonia This was also confirmed in this study as COPD patients with pneumonia had a longer hospital stay, and 15.9% of them were mechanically ventilated *vs*. 3% only in COPD patients without pneumonia.

By using Multivariate logistic regression analysis for readmission in COPD patients with or without CAP, it was found that length of hospital stay and C- reactive protein were significant causes with readmission and this was stated in Jing study that systemic inflammatory marker as C- reactive protein was a good predictor for readmission than sputum inflammatory markers [19] and confirmed in Rinne study stated that longer length of hospital stay for COPD hospitalizations was accompanied with a higher risk for readmission [20].

## CONCLUSION

COPD patients with CAP had a longer length of hospital stay and more short term complications as intensive care unit admission, invasive mechanical ventilation and higher readmission rate in comparison to COPD patients without CAP. Thus, prevention of CAP is essential to avoid the significant clinical and economic burden of illness in patients with COPD, especially in developing countries.

## Figures and Tables

**Table 1:** Characteristics of the patients between AECOPD with and without CAP.

-		**COPD without CAP Group** **(No,=100)**		**COPD with CAP Group** **(No,=90)**		**Chi square test/**
						**p value**
Age	Mean ±SD	64 ±8		62±12.		0.098
Pack/yr	Mean ±SD	88.90±38.60		87.84±41.73		0.857
Length of hospital stay(days)	Mean ±SD	7.57±2.24		11.30±3.23		<0.001**
COPD Assessment Test (CAT)	Mean± SD	28.95±6.01		28.56±5.72		0.647
mMRC Dyspnea scale	Mean± SD	2.91±0.64		2.95±0.48		0.592
-		**No**	**%**	**No**	**%**	**Independent t test**
						**p value**
LTOT	No	87	87.0%	76	84.4%	0.614
	Yes	13	13.0%	14	15.6%	
Comorbidities	Congestiveheart failure	20	20.0%	18	20.0%	0.057
	Chronickidney disease	5	5.0%	10	11.1%	
	DM	25	25.0%	26	28.9%	
	No	50	50.0%	36	40.0%	
MedicationBefore Admission	LCS	28	28.0%	8	9.1%	0.123
	LABA	18	18.0%	5	5.7%	
	SABA	2	2.0%	0	0.0%	
	LAMA	10	10.0%	3	3.4%	
	LCS+ LABA	42	42.0%	30	33.3%	

AECOPD: acute exacerbations of chronic obstructive pulmonary disease; CAP: community-acquired pneumonia; CAT: chronic obstructive pulmonary disease assessment test;; mMRC: modified Medical Research Council; n: number; SD: standard deviation; LTOT: long term oxygen therapy; ;**: P < 0.01: Highly significant (HS).

**Table 2:** Laboratory findings between AECOPD with and without CAP.

-		**COPD without CAP Group** **(N=100)**		**COPD with CAP Group** **(N=90)**		**Independent t Test**
		**Mean**	**SD**	**Mean**	**SD**	**p value**
Arterial Blood Gas	PH	7.03	0.17	7.09	0.29	0.070
	PCO2(mmHg)	48.11	9.97	47.02	8.66	0.432
	PO2(mmHg)	58.66	10.67	58.07	7.41	0.667
	SO2 (%)	92.44	6.24	92.35	4.53	0.911
Laboratory Investigation	BUN(mg/dL)	30.51	2.14	31.10	5.40	0.314
	Creatinne(mg/dL)	1.18	0.37	1.36	0.86	0.058
	Albumin(g/dl)	3.42	0.57	3.30	0.70	0.210
	Total Lecukocytecount(x12/L)	10.00	4.31	13.25	6.34	<0.001**
	Hemoglobin(g/dl)	13.38	2.02	12.65	2.17	0.018*
	Platelet(x12/L)	252.67	86.69	271.82	114.07	0.194
	CRP(mg/L)	9.68	1.65	13.64	2.54	0.001*
	Sodium(mmole/L)	137.61	2.86	137.03	4.20	0.263
	Potassium (mmole/L)	3.96	0.53	3.85	0.56	0.166

AECOPD: acute exacerbations of chronic obstructive pulmonary disease; BUN: blood urea nitrogen; CAP: community-acquired pneumonia; CRP: C-reactive protein; n: number; PaCO2: arterial carbon dioxide partial pressure; PaO2: arterial oxygen partial pressure; SD: standard deviation; SO2: oxygen saturation; *: p < 0.05: Significant (S); **: p < 0.01: Highly significant (HS).

**Table 3:** Comparison between COPD without CAP and COPD with CAP as regard complications.

**Complications**	**COPD without CAP Group** **(No=100)**		**COPD with CAP Group** **(No=90)**		***Chi* square test**
	**N**	**%**	**N**	**%**	**p value**
ICU admission	13	13.0%	41	45.6%	<0.001**
Mechanical ventilation	3	3.0%	14	15.9%	0.002*

AECOPD: acute exacerbations of chronic obstructive pulmonary disease; CAP: community-acquired pneumonia; n: Number; ICU: intensive care unit ;*: p < 0.05: Significant (S); **: p < 0.01: Highly significant (HS).

**Table 4:** Comparison between COPD without CAP Group and COPD with CAP Group as regard readmission.

-	**COPD without CAP Group** **(N=100)**		**COPD with CAP Group** **(N=90)**		***Chi* square test**
	**N**	**%**	**N**	**%**	**p value**
Readmission	27	27.0%	47	52.2%	0.003*

N: number; ;*: p < 0.05: Significant (S).

**Table 5:** Multivariate logistic regression analysis for readmission in COPD patients without CAP.

-	**B**	**S.E.**	**Wald**	**Sig.**	**Odds ratio ** **(OR)**	**95% C.I.for OR**	
						**Lower**	**Upper**
Length of hospital stay(days)	0.459	0.158	2.912	0.004*	0.016	0.146	0.772
Total Lecukocytecount(x12/L)	0.080	0.112	0.713	0.477	0.024	-0.142	0.302
Hemoglobin(g/dl)	0.577	0.300	1.921	0.058	0.011	-0.019	1.173
CRP(mg/L)	0.293	0.068	4.308	0.001*	0.009	0.158	0.428
ICU admission	0.239	0.701	0.116	0.733	0.788	0.199	3.109
mechanical ventilation	20.250	23.423	0.000	0.999	0.959	0.914	1.006

AECOPD: acute exacerbations of chronic obstructive pulmonary disease; CAP: community-acquired pneumonia; n: number ;CRP: C-reactive protein ; ICU: intensive care unit. ;*: p < 0.05: Significant (S); **: p < 0.01: Highly significant (HS).

**Table 6:** Multivariate logistic regression analysis for readmission in COPD patients with CAP.

-	**B**	**S.E.**	**Wald**	**Sig.**	**Odds ratio ** **(OR)**	**95% C.I.for OR**	
						**Lower**	**Upper**
Length of hospital stay(days)	0.776	0.192	4.048	0.001**	0.010	0.395	1.158
Total Lecukocytecount(x12/L)	0.672	0.124	5.420	0.001**	0.020	0.426	0.919
Hemoglobin(g/dl)	0.067	0.313	0.214	0.831	0.013	-0.554	0.688
CRP(mg/L)	0.544	0.086	6.333	0.001**	0.010	0.373	0.714
ICU admission	1.426	0.453	9.889	0.002*	4.162	1.711	10.123
mechanical ventilation	1.405	0.691	4.136	0.042*	4.074	1.052	15.773

AECOPD: acute exacerbations of chronic obstructive pulmonary disease; CAP: community-acquired pneumonia; n: number ; CRP: C-reactive protein ; ICU: intensive care unit. ;*: p < 0.05: Significant (S); **: p < 0.01: Highly significant (HS).

## LIST OF ABBREVIATIONS

AECOPD: Acute Exacerbation of Chronic Obstructive Pulmonary Disease Patients
CAP: Community-acquired Pneumonia
COPD: Chronic Obstructive Pulmonary Disease
